# 1,3-Dicyclo­hexyl­imidazolidine-2,4,5-trione: a second polymorph

**DOI:** 10.1107/S1600536812043619

**Published:** 2012-10-27

**Authors:** Oualid Talhi, José A. Fernandes, Diana C. G. A. Pinto, Artur M. S. Silva, Filipe A. Almeida Paz

**Affiliations:** a Department of Chemistry, University of Aveiro, QOPNA, 3810-193 Aveiro, Portugal; b Department of Chemistry, University of Aveiro, CICECO, 3810-193 Aveiro, Portugal

## Abstract

The title compound, C_15_H_22_N_2_O_3_, was obtained as a by-product of oxidative cleavage of 1,3-dicyclo­hexyl-(3-oxo-2,3-dihydro­benzofuran-2-yl)imidazolidine-2,4-dione. Herein, we report the crystal structure of a second polymorph, which was obtained by crystallization from an ethanol solution at 253 K, instead of slow evaporation of the same solvent at room temperature. While the first polymorph [Talhi *et al.* (2011). *Acta Cryst.* E**67**, o3243] crystallized in the non-centrosymmetric space group *P*2_1_2_1_2_1_, this second polymorph crystallizes in the centrosymmetric space group *P*2_1_/*n*. Compared to the first polymorph, in the crystal no C=O⋯C=O inter­actions were found (C⋯O inter­molecular distance longer than 3.15 Å) and instead, close packing of individual mol­ecular units is mediated by C—H⋯π and weak C—H⋯O inter­actions.

## Related literature

For the structure of the ortho­rhom­bic polymorph and further background information to the study, see: Talhi *et al.* (2011 ▶). For general background on crystallographic studies by our research group of related compounds having biological activity, see: Fernandes *et al.* (2011 ▶); Loughzail *et al.* (2011 ▶). For determination of the melting point, see: Ulrichan & Sayigh (1965 ▶).
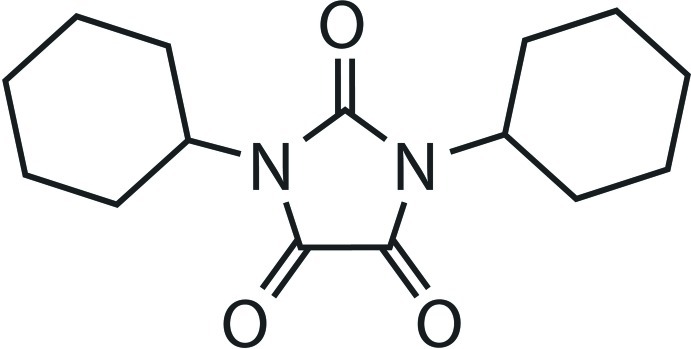


## Experimental

### 

#### Crystal data

C_15_H_22_N_2_O_3_
*M* *_r_* = 278.35
Monoclinic, 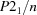

*a* = 5.1980 (2) Å
*b* = 21.7123 (10) Å
*c* = 13.0244 (6) Å
β = 100.163 (2)°
*V* = 1446.88 (11) Å^3^
*Z* = 4
Mo *K*α radiation
μ = 0.09 mm^−1^
*T* = 150 K
0.13 × 0.06 × 0.06 mm

#### Data collection

Bruker X8 Kappa CCD APEXII diffractometer
Absorption correction: multi-scan (*SADABS*; Sheldrick, 1998 ▶) *T*_min_ = 0.989, *T*_max_ = 0.995
38115 measured reflections
3876 independent reflections
2999 reflections with *I* > 2σ(*I*)
*R*_int_ = 0.049

#### Refinement

*R*[*F* ^2^ > 2σ(*F* ^2^)] = 0.043
*wR*(*F* ^2^) = 0.104
*S* = 1.04
3876 reflections
181 parameters
H-atom parameters constrained
Δρ_max_ = 0.37 e Å^−3^
Δρ_min_ = −0.18 e Å^−3^

### 

Data collection: *APEX2* (Bruker, 2006 ▶); cell refinement: *SAINT-Plus* (Bruker, 2005 ▶); data reduction: *SAINT-Plus* (Bruker, 2005 ▶); program(s) used to solve structure: *SHELXTL* (Sheldrick, 2008 ▶); program(s) used to refine structure: *SHELXTL* (Sheldrick, 2008 ▶); molecular graphics: *DIAMOND* (Brandenburg, 2009 ▶); software used to prepare material for publication: *SHELXTL* (Sheldrick, 2008 ▶).

## Supplementary Material

Crystal structure: contains datablock(s) global, I. DOI: 10.1107/S1600536812043619/nk2188sup1.cif

Structure factors: contains datablock(s) I. DOI: 10.1107/S1600536812043619/nk2188Isup2.hkl

Supplementary material file. DOI: 10.1107/S1600536812043619/nk2188Isup3.cdx

Supplementary material file. DOI: 10.1107/S1600536812043619/nk2188Isup4.cml

Additional supplementary materials:  crystallographic information; 3D view; checkCIF report

## Figures and Tables

**Table 1:** Short intermolecular interactions (Å, °) *Cg* is the centroid of the N1/N2/C1–C3 ring.

*D*—H⋯*A*	*D*—H	H⋯*A*	*D*⋯*A*	*D*—H⋯*A*
C7—H7*B*⋯*Cg* ^i^	0.99	2.78	3.5511 (14)	135
C11—H11*B*⋯O2^ii^	0.99	2.51	3.2065 (18)	127

Symmetry codes: (i) 
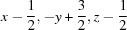
; (ii) 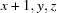.

**Fig. 1.**
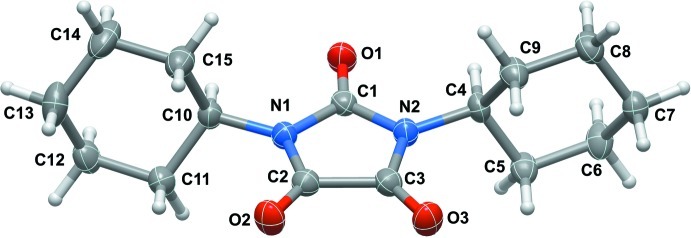
Schematic representation of the asymmetric unit of the title compound which comprises a whole molecule. Non-hydrogen atoms are represented as thermal ellipsoids drawn at the 70% probability level and hydrogen atoms as small spheres with arbitrary radii.

**Fig. 2.**
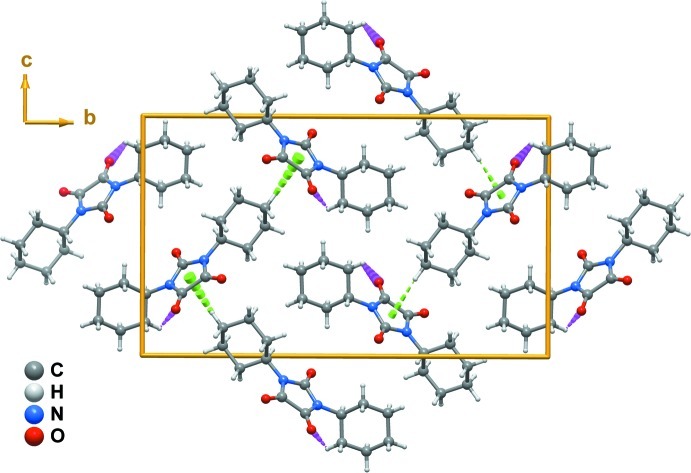
Crystal packing of the title compound viewed along the [100] direction of the unit cell. C—H···π interactions are represented as dashed green lines, and C—H···O weak hydrogen bonding interactions are represented as dashed pink lines. See Table 1 for geometrical details on the represented supramolecular interactions.

## supplementary crystallographic information

### Comment

In a previous publication (Talhi *et al.*, 2011) we described the crystal structure (polymorph I) of 1,3-dicyclohexylparabanic acid (see chemical diagram and Figure 1) obtained as a by-product of the oxidative cleavage of the C2'-C5 single bond of 1,3-dicyclohexyl-(3-oxo-2,3-dihydrobenzofuran-2-yl)imidazolidine-2,4-dione, using catalytic I_2_/DMSO system at 463 K. Following our interest on the structural features of compounds having biological activity (Fernandes *et al.*, 2011; Loughzail *et al.* 2011; Talhi *et al.*, 2011), particularly in our quest for novel polymorphic forms of pharmaceutic products, we wish to report the structure of a second crystalline polymorph of the title compound (polymorph II) obtained when applying a different crystallization procedure from that previously reported by us: while polymorph I was obtained by slow evaporation of an ethanolic solution at room temperature, polymorph II was obtained instead by cooling overnight the same solution at 253 K.

The asymmetric unit comprises a whole molecular unit of the title compound, C_15_H_22_N_2_O_3_ (Scheme and Figure 1). The central parabanic acid residue and the attached carbon atoms are coplanar with the largest deviation from the medium plane being 0.075 (1) Å for C4. The two cyclohexyl substituent groups appear exhibiting the chair typical conformation and their medium planes subtend slightly different angles with the aforementioned central plane, being one almost perpendicular [88.73 (5)°] and the other of 74.15 (6)°. We note that the observed angles for these two planes are larger than those registered for polymorph I in which the analogous values are *ca* 81 and 87° (Talhi *et al.*, 2011). Remarkably the four possible N—C—C—C groups involving three adjacent carbon atoms of the cyclohexyl moieties are also very near the planarity [largest deviation of 0.019 (1) Å for C10 in N1—C10—C11—C12].

The crystal packing is mainly governed by the need to fill the available space. A handful of weak supramolecular interactions are also observed, namely C—H···π and C—H···O (See Table 1 and Figure 2). While in the crystal structure of polymorph I a strong C═O···C═O interaction with a C···O distance smaller than 2.871 Å was observed, in the present polymorph II the shortest C···O intermolecular distance is 3.1519 (15) Å, which, in comparison to the case of polymorph I, may be considered as negligible.

### Experimental

The title compound was prepared following the procedure described previously (Talhi *et al.*, 2011), except for the crystallization process in which the raw compound was dissolved in ethanol and crystallized at 253 K overnight.

The melting point was measured on a Buchi B-540 equipment. NMR spectra were recorded on a Bruker Avance 300 spectrometer (300.13 for ^1^H and 75.47 MHz for ^13^C), with CDCl_3_ used as solvent. Chemical shifts (δ) are reported in p.p.m. and coupling constants (*J*) in Hz. The internal standard was TMS.

Unequivocal ^13^C assignments have been performed with the aid of bidimensional experiments (HSQC and HMBC). Both ^1^H and ^13^C NMR spectra show bilateral symmetry of the compound in solution. The HSQC spectrum allowed to deduce the electronegative effect of the nitrogen atom on the cyclohexyl proton and carbon resonances. However, it was found that the anisotropic effects of the carbonyl groups influence greatly the chemical shift values of the cyclohexyl proton resonances. Both of the highlighted effects of the parabanic nucleus heteroatoms are spread throughout the cyclohexyl chair skeleton decreasing gradually from C-1' to C-4'. Important features are recorded in the HMBC experiment concerning the carbon neighboring of the tertiary proton H-1' which correlates with the carbonyl groups C-2 and C-5, and further with C-2' and C-3' of the cyclohexyl radical.

Elemental Analysis: Calculated (in %): C, 64.73; H, 7.97; N, 10.06; Found: C, 64.47; H, 7.96; N, 10.05.

Melting point: 175 °C (Lit [Ulrichan & Sayigh, 1965] 174–175 °C).

HRMS(ESI+): *m/z* Calcd for [C_15_H_22_N_2_O_3_ + Na]^+^: 301.1528; found 301.1523.

^1^H NMR (300.13 MHz, CDCl_3_): δ = 1.14–1.26 and 1.63–1.71 (2 m, 4 H, H-4'), 1.26–1.43 and 1.80–1.91 (2 m, 4 H, H-3'), 1.73–1.77 and 1.97–2.19 (2 m, 4 H, H-2'), 4.00 (tt, *J*= 12.0 and 3.7 Hz, 2 H, H-1') p.p.m..

^13^C NMR (75.47 MHz, CDCl_3_): δ = 24.7 (C-4'), 25.6 (C-3'), 29.5 (C-2'), 52.4 (C-1'), 153.4 (C-2), 156.4 (C-4,5) p.p.m..

### Refinement

Hydrogen atoms bound to carbon were placed in idealized positions with C—H = 1.00 (for the tertiary carbons) and 0.99 Å (for the —CH_2_— moieties). These atoms were included in the final structural model in riding-motion approximation with the isotropic thermal displacement parameters fixed at 1.2×*U*_eq_ of the carbon atom to which they are attached.

### Crystal data

**Table:**

C_15_H_22_N_2_O_3_	*F*(000) = 600
*M**_r_* = 278.35	*D*_x_ = 1.278 Mg m^−^^3^
Monoclinic, *P*2_1_/*n*	Melting point: 175 K
Hall symbol: -P 2yn	Mo *K*α radiation, λ = 0.71073 Å
*a* = 5.1980 (2) Å	Cell parameters from 3876 reflections
*b* = 21.7123 (10) Å	θ = 3.7–29.1°
*c* = 13.0244 (6) Å	µ = 0.09 mm^−^^1^
β = 100.163 (2)°	*T* = 150 K
*V* = 1446.88 (11) Å^3^	Block, yellow
*Z* = 4	0.13 × 0.06 × 0.06 mm

### Data collection

**Table:**

Bruker X8 Kappa CCD APEXII diffractometer	3876 independent reflections
Radiation source: fine-focus sealed tube	2999 reflections with *I* > 2σ(*I*)
Graphite monochromator	*R*_int_ = 0.049
ω and φ scans	θ_max_ = 29.1°, θ_min_ = 3.7°
Absorption correction: multi-scan (*SADABS*; Sheldrick, 1998)	*h* = −6→7
*T*_min_ = 0.989, *T*_max_ = 0.995	*k* = −29→29
38115 measured reflections	*l* = −17→17

### Refinement

**Table:**

Refinement on *F*^2^	Primary atom site location: structure-invariant direct methods
Least-squares matrix: full	Secondary atom site location: difference Fourier map
*R*[*F*^2^ > 2σ(*F*^2^)] = 0.043	Hydrogen site location: inferred from neighbouring sites
*wR*(*F*^2^) = 0.104	H-atom parameters constrained
*S* = 1.04	*w* = 1/[σ^2^(*F*_o_^2^) + (0.0374*P*)^2^ + 0.6588*P*] where *P* = (*F*_o_^2^ + 2*F*_c_^2^)/3
3876 reflections	(Δ/σ)_max_ = 0.001
181 parameters	Δρ_max_ = 0.37 e Å^−^^3^
0 restraints	Δρ_min_ = −0.18 e Å^−^^3^

### Special details

**Table:**

Geometry. All e.s.d.'s (except the e.s.d. in the dihedral angle between two l.s. planes) are estimated using the full covariance matrix. The cell e.s.d.'s are taken into account individually in the estimation of e.s.d.'s in distances, angles and torsion angles; correlations between e.s.d.'s in cell parameters are only used when they are defined by crystal symmetry. An approximate (isotropic) treatment of cell e.s.d.'s is used for estimating e.s.d.'s involving l.s. planes.
Refinement. Refinement of *F*^2^ against ALL reflections. The weighted *R*-factor *wR* and goodness of fit *S* are based on *F*^2^, conventional *R*-factors *R* are based on *F*, with *F* set to zero for negative *F*^2^. The threshold expression of *F*^2^ > σ(*F*^2^) is used only for calculating *R*-factors(gt) *etc*. and is not relevant to the choice of reflections for refinement. *R*-factors based on *F*^2^ are statistically about twice as large as those based on *F*, and *R*- factors based on ALL data will be even larger.

### Fractional atomic coordinates and isotropic or equivalent isotropic displacement parameters (Å^2^)

**Table:**

	*x*	*y*	*z*	*U*_iso_*/*U*_eq_	
C1	0.9734 (2)	0.59882 (5)	0.12018 (9)	0.0179 (2)	
C2	0.7011 (2)	0.59707 (6)	0.23971 (10)	0.0187 (2)	
C3	0.6479 (2)	0.65531 (6)	0.17166 (10)	0.0189 (2)	
C4	0.8265 (2)	0.69411 (5)	0.01391 (9)	0.0182 (2)	
H4	0.9641	0.6788	−0.0245	0.022*	
C5	0.9020 (3)	0.75914 (6)	0.05202 (10)	0.0225 (3)	
H5A	1.0763	0.7585	0.0973	0.027*	
H5B	0.7740	0.7748	0.0937	0.027*	
C6	0.9077 (3)	0.80176 (6)	−0.04117 (11)	0.0246 (3)	
H6A	0.9480	0.8442	−0.0157	0.030*	
H6B	1.0482	0.7883	−0.0785	0.030*	
C7	0.6476 (3)	0.80154 (6)	−0.11652 (10)	0.0224 (3)	
H7A	0.5098	0.8190	−0.0815	0.027*	
H7B	0.6613	0.8278	−0.1775	0.027*	
C8	0.5724 (3)	0.73639 (6)	−0.15326 (10)	0.0255 (3)	
H8A	0.7001	0.7208	−0.1951	0.031*	
H8B	0.3982	0.7371	−0.1986	0.031*	
C9	0.5654 (3)	0.69293 (6)	−0.06145 (10)	0.0235 (3)	
H9A	0.4236	0.7056	−0.0242	0.028*	
H9B	0.5282	0.6505	−0.0877	0.028*	
C10	1.0256 (2)	0.50852 (5)	0.24346 (10)	0.0185 (2)	
H10	1.1926	0.5048	0.2158	0.022*	
C11	1.0949 (3)	0.50699 (6)	0.36255 (10)	0.0227 (3)	
H11A	0.9339	0.5107	0.3929	0.027*	
H11B	1.2105	0.5421	0.3876	0.027*	
C12	1.2335 (3)	0.44639 (6)	0.39731 (11)	0.0257 (3)	
H12A	1.4008	0.4443	0.3711	0.031*	
H12B	1.2733	0.4448	0.4744	0.031*	
C13	1.0635 (3)	0.39147 (6)	0.35622 (13)	0.0334 (3)	
H13A	0.9026	0.3916	0.3871	0.040*	
H13B	1.1593	0.3528	0.3773	0.040*	
C14	0.9900 (3)	0.39390 (6)	0.23745 (13)	0.0351 (4)	
H14A	0.8732	0.3589	0.2129	0.042*	
H14B	1.1499	0.3897	0.2065	0.042*	
C15	0.8525 (3)	0.45432 (6)	0.20050 (11)	0.0263 (3)	
H15A	0.8176	0.4558	0.1233	0.032*	
H15B	0.6832	0.4569	0.2251	0.032*	
N1	0.9031 (2)	0.56747 (5)	0.20531 (8)	0.0185 (2)	
N2	0.8172 (2)	0.65170 (5)	0.10196 (8)	0.0182 (2)	
O1	1.13883 (18)	0.58316 (4)	0.07174 (7)	0.0244 (2)	
O2	0.58424 (18)	0.58220 (4)	0.30792 (7)	0.0260 (2)	
O3	0.49016 (18)	0.69488 (4)	0.18021 (8)	0.0259 (2)	

### Atomic displacement parameters (Å^2^)

**Table:**

	*U*^11^	*U*^22^	*U*^33^	*U*^12^	*U*^13^	*U*^23^
C1	0.0198 (6)	0.0174 (5)	0.0157 (6)	−0.0008 (4)	0.0015 (5)	0.0002 (4)
C2	0.0183 (6)	0.0194 (5)	0.0182 (6)	−0.0008 (4)	0.0023 (5)	−0.0007 (4)
C3	0.0186 (6)	0.0201 (6)	0.0178 (6)	−0.0009 (5)	0.0025 (5)	0.0005 (5)
C4	0.0201 (6)	0.0183 (5)	0.0160 (6)	0.0007 (4)	0.0031 (5)	0.0049 (4)
C5	0.0232 (7)	0.0206 (6)	0.0215 (6)	−0.0030 (5)	−0.0022 (5)	0.0030 (5)
C6	0.0228 (7)	0.0216 (6)	0.0283 (7)	−0.0030 (5)	0.0012 (5)	0.0060 (5)
C7	0.0218 (6)	0.0223 (6)	0.0227 (6)	0.0025 (5)	0.0030 (5)	0.0060 (5)
C8	0.0282 (7)	0.0262 (6)	0.0198 (6)	−0.0003 (5)	−0.0019 (5)	0.0037 (5)
C9	0.0244 (7)	0.0227 (6)	0.0213 (6)	−0.0048 (5)	−0.0022 (5)	0.0024 (5)
C10	0.0209 (6)	0.0165 (5)	0.0182 (6)	0.0023 (4)	0.0041 (5)	0.0021 (4)
C11	0.0272 (7)	0.0213 (6)	0.0189 (6)	0.0027 (5)	0.0023 (5)	0.0016 (5)
C12	0.0277 (7)	0.0278 (6)	0.0210 (6)	0.0061 (5)	0.0026 (5)	0.0055 (5)
C13	0.0327 (8)	0.0228 (7)	0.0434 (9)	0.0033 (6)	0.0036 (7)	0.0134 (6)
C14	0.0391 (8)	0.0171 (6)	0.0442 (9)	0.0011 (6)	−0.0062 (7)	−0.0031 (6)
C15	0.0297 (7)	0.0187 (6)	0.0279 (7)	0.0001 (5)	−0.0025 (6)	−0.0008 (5)
N1	0.0214 (5)	0.0175 (5)	0.0176 (5)	0.0015 (4)	0.0059 (4)	0.0019 (4)
N2	0.0196 (5)	0.0176 (5)	0.0178 (5)	0.0020 (4)	0.0044 (4)	0.0027 (4)
O1	0.0287 (5)	0.0242 (4)	0.0225 (5)	0.0060 (4)	0.0105 (4)	0.0031 (4)
O2	0.0259 (5)	0.0288 (5)	0.0255 (5)	0.0017 (4)	0.0110 (4)	0.0057 (4)
O3	0.0251 (5)	0.0254 (5)	0.0284 (5)	0.0074 (4)	0.0079 (4)	0.0030 (4)

### Geometric parameters (Å, º)

**Table:**

C1—O1	1.2017 (15)	C8—H8A	0.9900
C1—N2	1.4022 (15)	C8—H8B	0.9900
C1—N1	1.4033 (15)	C9—H9A	0.9900
C2—O2	1.2052 (15)	C9—H9B	0.9900
C2—N1	1.3723 (16)	C10—N1	1.4760 (15)
C2—C3	1.5409 (17)	C10—C15	1.5262 (17)
C3—O3	1.2062 (15)	C10—C11	1.5296 (17)
C3—N2	1.3734 (16)	C10—H10	1.0000
C4—N2	1.4781 (15)	C11—C12	1.5297 (18)
C4—C5	1.5250 (17)	C11—H11A	0.9900
C4—C9	1.5284 (18)	C11—H11B	0.9900
C4—H4	1.0000	C12—C13	1.524 (2)
C5—C6	1.5307 (18)	C12—H12A	0.9900
C5—H5A	0.9900	C12—H12B	0.9900
C5—H5B	0.9900	C13—C14	1.527 (2)
C6—C7	1.5239 (18)	C13—H13A	0.9900
C6—H6A	0.9900	C13—H13B	0.9900
C6—H6B	0.9900	C14—C15	1.5302 (19)
C7—C8	1.5220 (18)	C14—H14A	0.9900
C7—H7A	0.9900	C14—H14B	0.9900
C7—H7B	0.9900	C15—H15A	0.9900
C8—C9	1.5288 (18)	C15—H15B	0.9900
			
O1—C1—N2	126.12 (11)	H9A—C9—H9B	108.1
O1—C1—N1	126.00 (11)	N1—C10—C15	110.74 (10)
N2—C1—N1	107.88 (10)	N1—C10—C11	111.77 (10)
O2—C2—N1	129.00 (12)	C15—C10—C11	111.92 (10)
O2—C2—C3	125.55 (11)	N1—C10—H10	107.4
N1—C2—C3	105.45 (10)	C15—C10—H10	107.4
O3—C3—N2	128.81 (12)	C11—C10—H10	107.4
O3—C3—C2	125.96 (11)	C10—C11—C12	109.47 (10)
N2—C3—C2	105.22 (10)	C10—C11—H11A	109.8
N2—C4—C5	111.47 (10)	C12—C11—H11A	109.8
N2—C4—C9	109.93 (10)	C10—C11—H11B	109.8
C5—C4—C9	111.89 (10)	C12—C11—H11B	109.8
N2—C4—H4	107.8	H11A—C11—H11B	108.2
C5—C4—H4	107.8	C13—C12—C11	110.82 (11)
C9—C4—H4	107.8	C13—C12—H12A	109.5
C4—C5—C6	109.97 (10)	C11—C12—H12A	109.5
C4—C5—H5A	109.7	C13—C12—H12B	109.5
C6—C5—H5A	109.7	C11—C12—H12B	109.5
C4—C5—H5B	109.7	H12A—C12—H12B	108.1
C6—C5—H5B	109.7	C12—C13—C14	110.77 (12)
H5A—C5—H5B	108.2	C12—C13—H13A	109.5
C7—C6—C5	111.69 (11)	C14—C13—H13A	109.5
C7—C6—H6A	109.3	C12—C13—H13B	109.5
C5—C6—H6A	109.3	C14—C13—H13B	109.5
C7—C6—H6B	109.3	H13A—C13—H13B	108.1
C5—C6—H6B	109.3	C13—C14—C15	111.56 (12)
H6A—C6—H6B	107.9	C13—C14—H14A	109.3
C8—C7—C6	110.79 (11)	C15—C14—H14A	109.3
C8—C7—H7A	109.5	C13—C14—H14B	109.3
C6—C7—H7A	109.5	C15—C14—H14B	109.3
C8—C7—H7B	109.5	H14A—C14—H14B	108.0
C6—C7—H7B	109.5	C10—C15—C14	109.49 (11)
H7A—C7—H7B	108.1	C10—C15—H15A	109.8
C7—C8—C9	111.59 (11)	C14—C15—H15A	109.8
C7—C8—H8A	109.3	C10—C15—H15B	109.8
C9—C8—H8A	109.3	C14—C15—H15B	109.8
C7—C8—H8B	109.3	H15A—C15—H15B	108.2
C9—C8—H8B	109.3	C2—N1—C1	110.60 (10)
H8A—C8—H8B	108.0	C2—N1—C10	127.27 (10)
C4—C9—C8	110.55 (11)	C1—N1—C10	121.98 (10)
C4—C9—H9A	109.5	C3—N2—C1	110.76 (10)
C8—C9—H9A	109.5	C3—N2—C4	126.14 (10)
C4—C9—H9B	109.5	C1—N2—C4	122.96 (10)
C8—C9—H9B	109.5		
			
O2—C2—C3—O3	3.1 (2)	O2—C2—N1—C10	1.1 (2)
N1—C2—C3—O3	−177.10 (12)	C3—C2—N1—C10	−178.71 (11)
O2—C2—C3—N2	−177.10 (12)	O1—C1—N1—C2	−177.91 (12)
N1—C2—C3—N2	2.67 (13)	N2—C1—N1—C2	2.28 (14)
N2—C4—C5—C6	−179.59 (10)	O1—C1—N1—C10	−1.95 (19)
C9—C4—C5—C6	−56.03 (14)	N2—C1—N1—C10	178.24 (10)
C4—C5—C6—C7	56.11 (15)	C15—C10—N1—C2	76.87 (15)
C5—C6—C7—C8	−56.18 (15)	C11—C10—N1—C2	−48.64 (16)
C6—C7—C8—C9	55.57 (15)	C15—C10—N1—C1	−98.38 (13)
N2—C4—C9—C8	−179.76 (10)	C11—C10—N1—C1	136.10 (12)
C5—C4—C9—C8	55.82 (14)	O3—C3—N2—C1	178.39 (13)
C7—C8—C9—C4	−55.24 (15)	C2—C3—N2—C1	−1.36 (13)
N1—C10—C11—C12	−177.09 (10)	O3—C3—N2—C4	−5.9 (2)
C15—C10—C11—C12	58.05 (14)	C2—C3—N2—C4	174.36 (11)
C10—C11—C12—C13	−57.32 (15)	O1—C1—N2—C3	179.78 (12)
C11—C12—C13—C14	57.00 (16)	N1—C1—N2—C3	−0.41 (14)
C12—C13—C14—C15	−56.42 (17)	O1—C1—N2—C4	3.89 (19)
N1—C10—C15—C14	177.51 (12)	N1—C1—N2—C4	−176.29 (10)
C11—C10—C15—C14	−57.06 (15)	C5—C4—N2—C3	64.30 (16)
C13—C14—C15—C10	55.77 (17)	C9—C4—N2—C3	−60.36 (15)
O2—C2—N1—C1	176.75 (13)	C5—C4—N2—C1	−120.46 (12)
C3—C2—N1—C1	−3.01 (13)	C9—C4—N2—C1	114.87 (13)

### Hydrogen-bond geometry (Å, º)

Cg is the centroid of the N1, N2, C1–C3 ring. <[C—H···(ring plane)] is *ca.* 43°.

**Table:**

*D*—H···*A*	*D*—H	H···*A*	*D*···*A*	*D*—H···*A*
C7—H7*B*···*Cg*^i^	0.99	2.78	3.5511 (14)	135
C11—H11*B*···O2^ii^	0.99	2.51	3.2065 (18)	127

Symmetry codes: (i) *x*−1/2, −*y*+3/2, *z*−1/2; (ii) *x*+1, *y*, *z*.
